# Sequence Recombination and Conservation of *Varroa* *destructor* *Virus-1* and *Deformed Wing Virus* in Field Collected Honey Bees (*Apis mellifera*)

**DOI:** 10.1371/journal.pone.0074508

**Published:** 2013-09-18

**Authors:** Hui Wang, Jiazheng Xie, Tim G. Shreeve, Jinmin Ma, Denise W. Pallett, Linda A. King, Robert D. Possee

**Affiliations:** 1 Centre for Ecology and Hydrology, Natural Environmental Research Council, Wallingford, Oxfordshire, United Kingdom; 2 Beijing Genome Institute, Yantian District, Shenzhen, China; 3 Department of Biological and Medical Sciences, Oxford Brooks University, Oxford, United Kingdom; Plymouth University, United Kingdom

## Abstract

We sequenced small (s) RNAs from field collected honeybees (*Apis mellifera*) and bumblebees ( *Bombus* *pascuorum* ) using the Illumina technology. The sRNA reads were assembled and resulting contigs were used to search for virus homologues in GenBank. Matches with *Varroa* *destructor* *virus-1* (VDV1) and Deformed wing virus (DWV) genomic sequences were obtained for *A. mellifera* but not *B* *. pascuorum* . Further analyses suggested that the prevalent virus population was composed of VDV-1 and a chimera of 5’-DWV-VDV1-DWV-3’. The recombination junctions in the chimera genomes were confirmed by using RT-PCR, cDNA cloning and Sanger sequencing. We then focused on conserved short fragments (CSF, size > 25 nt) in the virus genomes by using GenBank sequences and the deep sequencing data obtained in this study. The majority of CSF sites confirmed conservation at both between-species (GenBank sequences) and within-population (dataset of this study) levels. However, conserved nucleotide positions in the GenBank sequences might be variable at the within-population level. High mutation rates (Pi>10%) were observed at a number of sites using the deep sequencing data, suggesting that sequence conservation might not always be maintained at the population level. Virus-host interactions and strategies for developing RNAi treatments against VDV1/DWV infections are discussed.

## Introduction

Virus infections are closely associated with colony collapse in the European honeybee *Apis mellifera* (reviewed by [1,2]). Recent pandemics of colony collapse around the world have also been associated with invasion of honeybee populations by the mite *Varroa* *destructor* . Colonisation of hives by *V* *. destructor* in areas previously mite free has resulted in the selection of certain Deformed wing virus (DWV) strains [3-6]. The emergence of these selected strains appears to disturb the balance between the virus and honeybee populations, thus initiating collapse of honeybee colonies [3-7]. In areas where *V* *. destructor* is established, virus recombination occurs and chimeric viruses between DWV and the *Varroa* *destructor* *virus-1* (VDV1) [8-10] have been reported [10,11].

Both DWV [1,5,6,11-42] and VDV-1 [8-10] belong to the Iflaviridae (genus *Iflavirus*) [8,43], a picorna-like family of insect viruses. There is also a Kakugo virus (KV, genus *Iflavirus*) [44-46], which is very closely related to DWV genetically but is associated with aggressive behaviour in guard bees. These viruses have positive-sense, single-stranded RNA (+ssRNA) genomes of approximately 10 Kb, containing a single open reading frame flanked by 5’ and 3’ untranslated regions (UTR). The two viruses are phylogenetically related and form a DWV/VDV-1 clade with conserved amino acid motifs and a 3’-UTR [8]. As expected for picorna-like viruses, DWV populations display high mutation rates and quasi-species characteristics (reviewed by [25]). Chimeric viruses between DWV and VDV-1 have been reported in Israel [10] and the UK [11], suggesting that genome recombination between the two viruses may not be rare. In both cases, the chimera viruses co-persisted with one of the parents, either VDV-1 [10] or DWV [11]. In the latter case, two chimera viruses distinguishable by recombination patterns were detected [11]. The apparent unpredictable recombination pattern has added to the complexity of evolutionary trends in the DWV/VDV-1 group. Most importantly, rapid viral evolution represents an elevated challenge for developing treatments and/or controlling measures against the virus infections.

One of the new strategies to control viral diseases is RNA interfering (RNAi) technology (reviewed in 1). RNAi (also known as post-transcriptional gene silencing) is an ancient intracellular mechanism shared by both prokaryotes and eukaryotes and can mediate a specific anti-virus function (e.g., reviewed by [47,48]). In brief, host RNase III-like Dicer enzymes target double-stranded (ds) RNAs, such as viral RNA replication intermediates. A cascade of host enzyme actions is initiated to produce primary and secondary viral derived small interfering RNAs (vsiRNA) that specifically target the viral ssRNAs based on sequence complementary homology [49]. Based on their specific anti-virus function, vsiRNA sequencing has become a new technique for detecting unknown virus prevalence and discovering novel viruses, particularly from plant and insect materials [50-52]. High throughput next generation sequencing (NGS) technologies makes sequencing based approaches very efficient for virus survey and discovery [53-56]. Based on vsiRNAs’ virus inhibitory function, RNAi technology has also been developed to control virus infections [1,57] including within insects [58]. In honeybees, RNAi has been successfully employed against Israeli acute paralysis virus (IAPV) infections in both laboratory and large-scale trials [59,60]. Anti-IAPV immunity was introduced by feeding double-stranded viral RNA fragments to induce vsiRNA production. Feeding double-stranded DWV RNA fragments to honeybees has also been shown to reduce virus load, wing deformity and increase longevity when compared with mock treated controls [26].

In addition to honeybees, bumblebees (e.g. *Bombus* *pascuorum* ) are important wild pollinators [61] and cross-species virus infections have been reported for honeybees and wild pollinators including bumblebees, reflecting a scenario that viruses may be transmitted from wild pollinators to hive bees [61,62]. To investigate potential virus infections, we sequenced small RNA (sRNA) from extracts from *B* *. pascuorum* and *A. mellifera* collected from the field in Oxfordshire, UK. Small RNA deep sequencing was deployed to detect virus prevalence followed by validations using RT-PCR and Sanger sequencing. We also conducted a within-population analysis of single nucleotide polymorphism (SNP) using the deep sequencing dataset and analysed conserved short fragments (CSF) in the virus genomes to identify possible targets for RNAi development against infections of the VDV-1/DWV group of viruses that associate with collapse of honeybee colonies worldwide.

## Results

**Figure 1:**
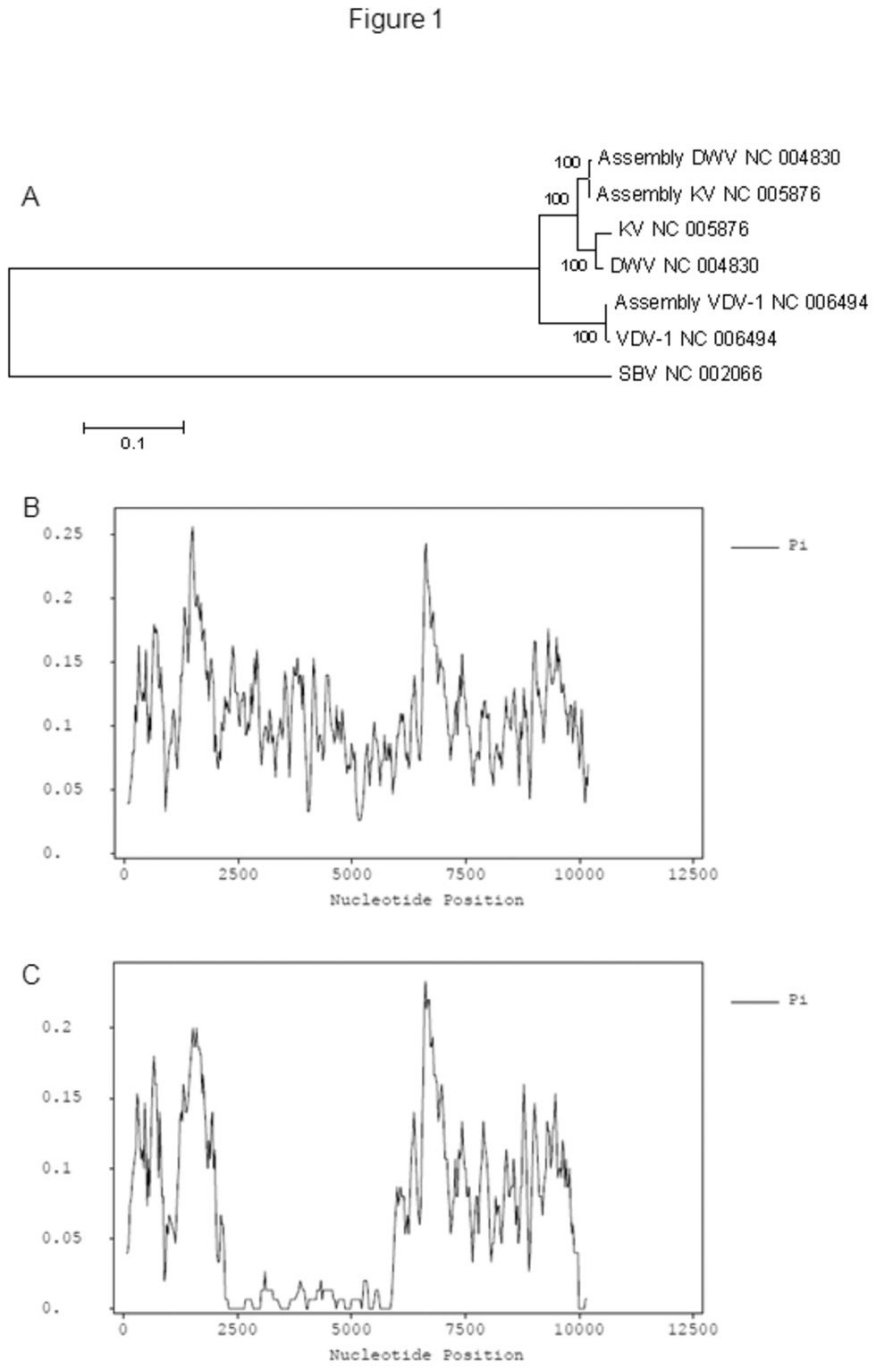
Comparison of assembled virus genomes. (A) A Neighbour Joint tree constructed with 1000 bootstraps. NCBI reference sequences were labelled with virus names followed by the GenBank accession numbers. The Maq assembled sequences were labelled accordingly to the reference sequences used. The *Sacbrood* *virus* (SBV) reference genome was used as an outer sequence. (B) SNP profile using the 3 viral reference sequences. (C) SNP profile using the 3 Maq assembled virus genomes.

**Figure 2:**
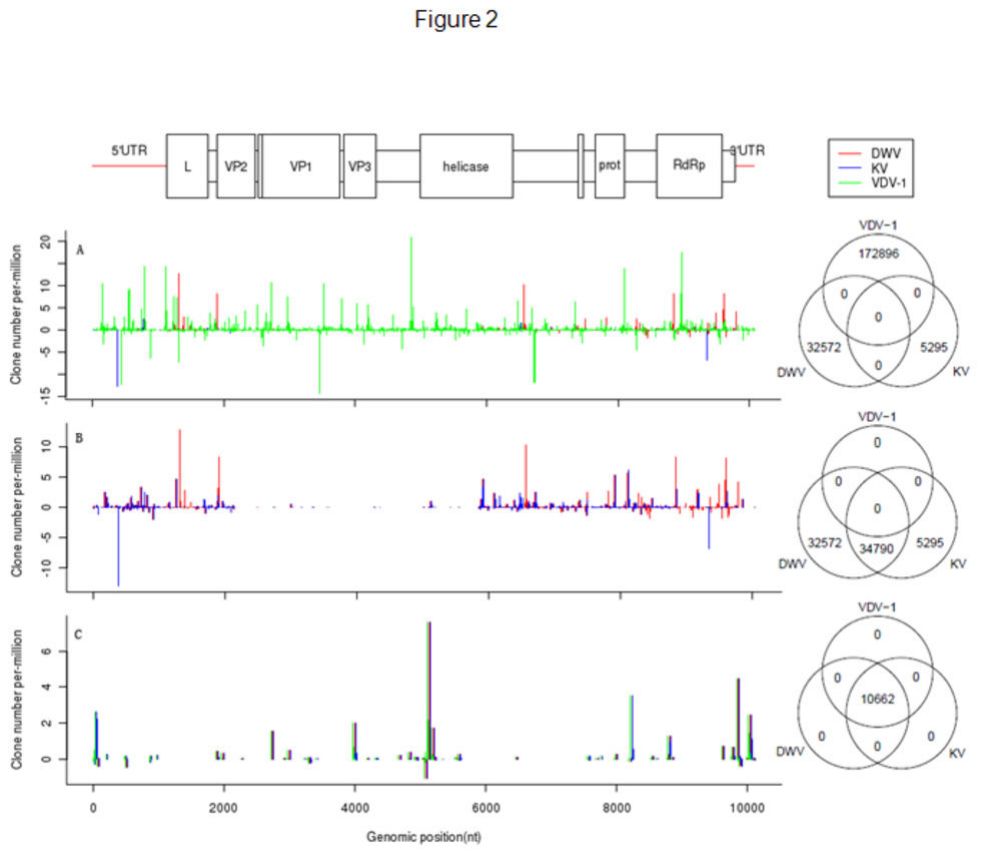
Virus derived small interfering (vsiRNA) mapping profiles. (A) Unique (non-redundant) vsiRNAs mapped to either VDV-1 or DWV or KV genomes. Shared reads were excluded. (B) Positions of vsiRNAs mapped to DWV and KV genomes. Shared reads with VDV-1 were excluded. (C) Shared vsiRNAs mapped to all of the three virus genomes. Note that the Y-axis represents both plus and minus strands and the scale is different among the three panels.

From the pooled honeybee and bumblebee sRNA extracts, approximately 95 million (M) high quality sRNA reads were obtained in total. There were 1.08 M unique reads ranging between 18-44 nt with the dominating species being 22-nt long Dicer products (Figure S1). After removal of ncRNAs (Table S1 and Figure S2), 3.77 M of total reads remained in the library containing 0.93 M unique reads (Figure S1). These unique reads were assembled firstly by the SOAPdenovo program and the resulting contigs were subjected to BLAST searches against virus reference genomes in the NCBI. Matches with three viruses (Text S1) were obtained with high scores. These were the Deformed wing virus (DWV), *Varroa* *destructor* *virus-1* (VDV1) and *Kakugovirus* (KV). A later result showed that these viral signals were only associated with the honeybee extracts. Viral genomes (NCBI, DWV, NC_004830; VDV-1, NC_006494; KV, NC_005876) were used as the references to enable MAQ assembly of the virus genome. The assembled consensus genomes (NCBI accession numbers: VDV-1_Ox, KC786222; DWV_Ox, KC786223; KVlike_Ox, KC786224) displayed close but distinguishable phylogenetic relationships (Figure 1), suggesting mixed infections in the sample. When sRNA reads were mapped against the reference sequences, 31,192 unique reads could be perfectly matched to either the VDV-1, WDV or KV genomes (Figure 2, Table S2 and Figure S3). These vsiRNAs were also dominated by 22-nt long species (Figure S1), distributed throughout the genomes with hotspots, and originated from both plus and minus RNA strands (Figure 2).

The vsiRNAs could be mapped through-out the whole VDV-1_Ox genome (Figure 2) but represented a gap against the DWV_Ox (2168-5853 nt) and KVlike_Ox (2164-5865 nt) genomes (Figure 2). This gap was located between the VP2 encoding region and helicase encoding region, and did not appear to be due to sequence similarities among the VDV-1, DWV and KV genomes (Figure 2). Sequence conservations among the three viruses resulted in a few vsiRNA islands being shared by these viruses (Figure 2). SNP analysis using the virus reference genomes (Figure 1) and the MAQ assembled genomes confirmed this conclusion (Figure 1). The absence of DWV/KV sequences in the middle fractions suggested that the VP2-Helicase fragment of DWV_Ox/KVlike_Ox was replaced by the sequences of VDV-1_Ox.

**Figure 3:**
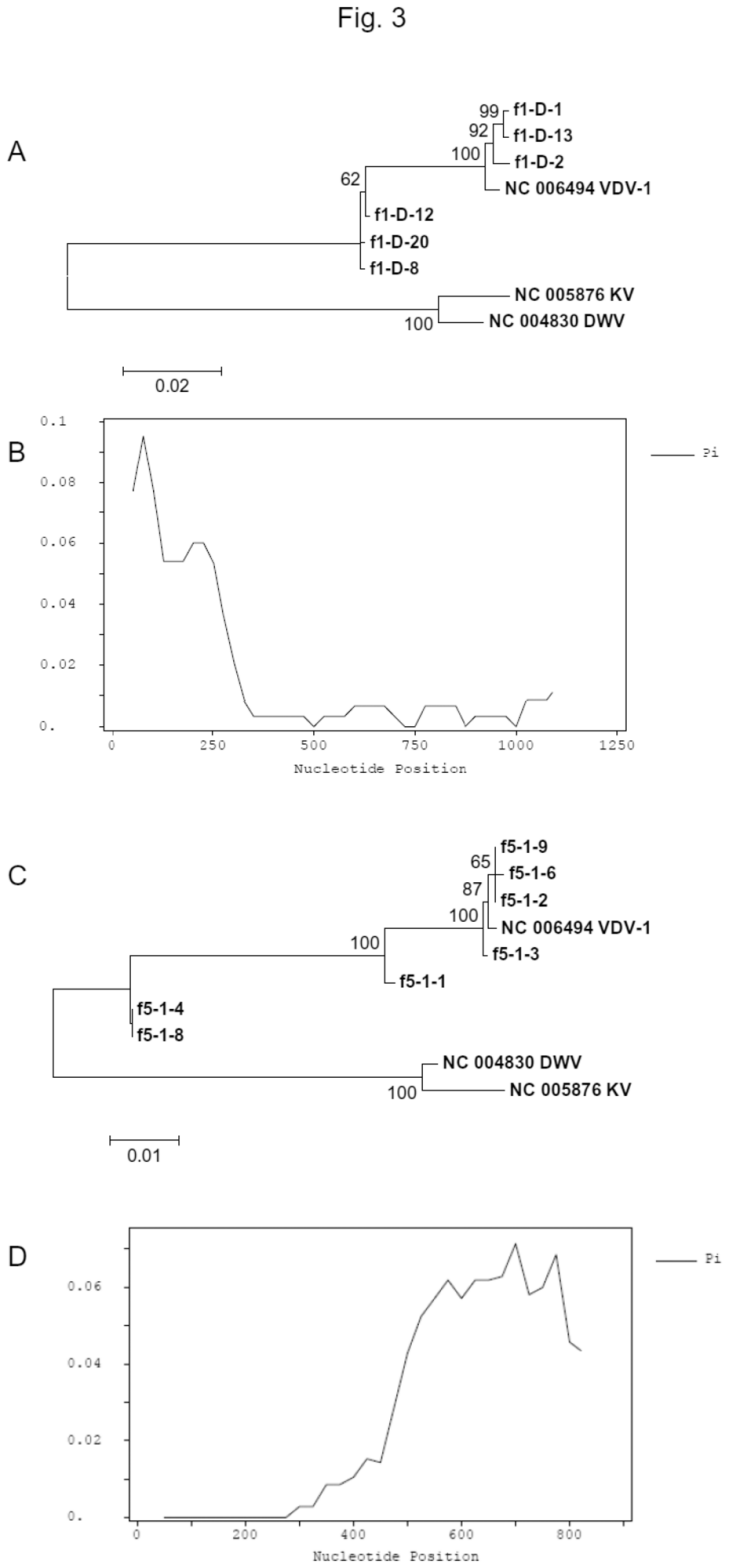
Sanger sequencing validation of chimera virus. (A) Unrooted Neighbour Joint tree of the 5’ recombination junction using the sequences of RT-PCR products amplified by primers F1R1. The tree was constructed with 1000 bootstraps and scores higher than 50% were displayed. Reference sequences are labelled with the NCBI accession numbers followed by virus names. The Sanger sequences obtained in this work (NCBI Accession No: KC691296-KC691301) are labelled as F1 followed by in-house sequence IDs. (B) SNP profile of the 5’ recombination junction using the Sanger sequences, showing a decrease of polymorphism after the recombination junction. (C) Unrooted Neighbour Joint tree of the 3’ recombination junction using the sequences of RT-PCR products amplified by primers F5R5. The tree was constructed with 1000 bootstraps and scores higher than 50% are displayed. Reference sequences are labelled with the NCBI accession numbers followed by virus names. The Sanger sequences obtained in this work (GenBank Accession No: KC691302-KC691308) are labelled as F5 followed by in-house sequence IDs. (D) SNP profile of the 3’ recombination junction using the Sanger sequences, showing that nucleotide SNPs increased after the recombination junction.

To confirm that the viral population was a mixture of a VDV-1 virus and a 5’ DWV-VDV1-DWV3’ chimera, we amplified the putative recombination junction regions from the total RNA extract by using RT-PCR. The honeybee RNA extracts produced single band products at both nt 1874-3001 (VDV-1) and nt 5386-6252 (VDV-1) regions, whereas RNA extracts of the bumblebee were negative (Figure S4), suggesting that the local infection might be limited in honeybees although DWV and VDV-1 infections have been reported in bumblebees [28,32,62,69]. Negative bumblebee samples also demonstrated that there was no viral contamination during sample processing. The RT-PCR products were cloned and then sequenced using the Sanger method. Phylogenetic trees of sequences in these two fragments clearly showed that there were mixed infections (Figure 3). SNP analyses using the Sanger sequences confirmed the sRNA assembly and mapping result (Figure 1, Figure 2) that sequences in the nt 2100-5800 nt region (Figure 2) contained much less polymorphism than the 5’- and 3’-fragments (Figure 3, Text S2, Text S3), the presence of chimera virus and that the sRNA deep sequencing result was genuine rather than an artefact due to unexpected sequencing bias.

**Figure 4:**
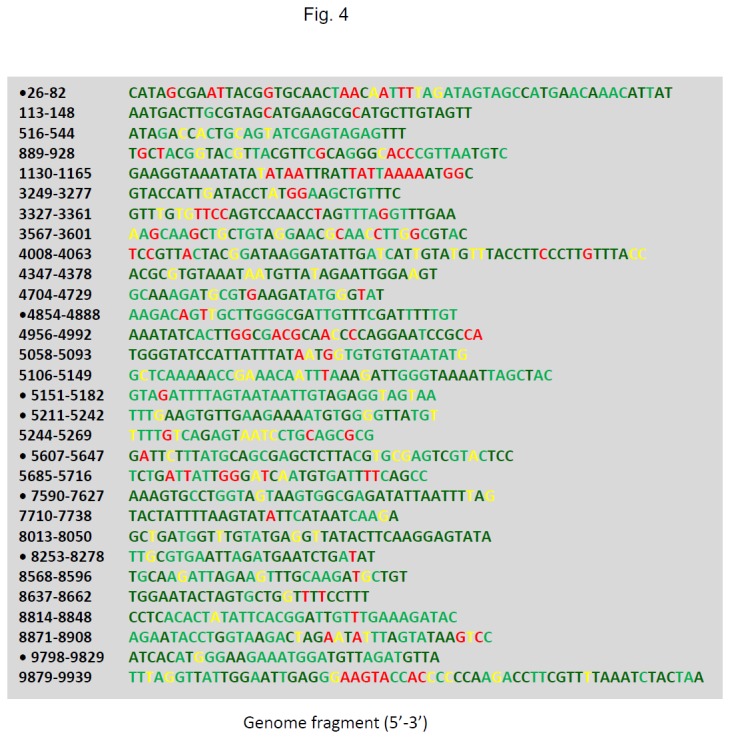
Conserved short fragments (CSF) of the virus population. Between-species conserved regions (consecutively larger than 25 nt, without any SNP) were identified by using sequence alignment of the VDV-1/DWV/KV clade (Figure S5). Within-population SNP was calculated by using the sRNA deep sequencing dataset. Each CSF was labelled using DWV genome positions. Colour coded positions represented within-population mutation rate (Pi) as Pi = 0 (dark green), Pi < 5% (light green), 5% ≤ Pi < 10% (yellow) and Pi ≥ 10% (red). The mutation rate at each nucleotide position was reported in Table S3. Black dots label CSFs with 20 consecutive low Pi sites at the within-population level.

Deep sequencing technology provides new opportunities to analyse mutation profiles at each nucleotide position of a virus genome. Here we focused on the conserved positions. Reads were mapped against the reference sequences with 2-nt mismatches allowed. A mutation profile of the infecting virus population was obtained (Data not shown). To further evaluate the observation, we downloaded all *Iflavirus* sequences from the GenBank (by Dec 2012, Figure S5). These GenBank reference sequences were aligned and conserved regions were screened by using sliding windows (Window size 25-nt) in Program DnaSP [67]. Thirty conserved short fragments (CSF) were observed (Figure 4 and Table S3) from the sequences of the VDV-1/DWV/KV clade (Figure S5). The largest CSF was 61-nt long and located at the 3’-end of the virus genome (Figure 4). Using the sRNA library, mutation rates (Pi) at each nucleotide positions were calculated and they were not correlated to sequencing coverage in these CSF (Figure S6), indicating that the SNPs observed in the deep sequencing dataset were not largely due to sequencing error which could be expected at a rate of 0.02% with the Illumina Solexa technology. Although these sites appeared conserved at the between-species level, significant SNP (e.g., Pi > 10%) was observed for some of the positions at the within-population level (Figure 4, Figure S6, Table S3). However, the majorities of the CSF sites did display low (e.g. Pi < 5%) or nil SNP (Figure 4). The observed high mutation rate (Figure 4) is indicative that nucleotide constraint may not be initiated at the within-population level. There were eight CSFs having a consistent 20-nt long region (Figure 4) in which the observed mutation rates were lower than 5%.

## Discussion

RNAi is an ancient anti-viral mechanism. The vsiRNAs generated by host RNase III enzymes are integrated with host Argonaut (AGO) enzymes and lead the RISC (RNA-Induced Silencing Complex) to target viral single-stranded RNAs based on complementary sequence homology. In turn, viruses have evolved suppressor proteins against host RNAi (reviewed by [70]). On the other hand, due to error prone RNA dependent RNA polymerase, RNA viruses have high mutation rates and their populations display quasi-species characteristics. It is well known that animal viruses may mutate to escape host antibody derived immunities (reviewed by [71]). Regarding host RNAi immunity (reviewed by [47,48]), viruses may also evolve to change their genome composition to reduce host RNAi response during infections [72-74]. Single nucleotide mutations were not considered as a feasible evolutionary strategy for viruses to combat the natural host RNAi because RNAi targets the whole of the virus genome simultaneously (e.g. Figure 2), making it impossible for the virus to deploy sufficient site mutations to escape. Human introduced anti-virus RNAi immunity, however, often targets a small region in the virus genome, allowing the virus to accumulate sufficient mutations in the targeted region [75,76]. It therefore becomes necessary to develop strategies to minimise the chances of virus escape by mutation. A logical approach is to target conserved viral sequences in RNAi development. This is because conserved sequences are likely to have important functions constrained by strong negative evolutionary selection. We have identified eight CSF that were relatively constrained at both within-species and between-species levels (Figure 4). These are likely to be functionally important motifs for virus survival and it would be unlikely that the virus could generate escape mutations if they were targeted. To obtain a comprehensive CSF profile of the VDV-1/DWV/KV clade viruses, spatially distinct virus populations need to be investigated.

Iflaviruses have diverse population structures that can shift dynamically according to selection pressure and environmental changes (e.g., [6]). Between-species genome recombination and chimera virus infections have been reported for the DWV/VDV-1 group [10,11]. By comparing sequences deposited in public databases, the research community builds up a capacity to determine conserved regions of virus genomes. Although codon constraint [77] and RNA folding structure [78] have been well considered, knowledge about function and maintenance of conserved nucleotide positions in virus genomes is still limited. It may be logical to assume that such sequence conservation is maintained at the very basic level, i.e., within-population level. By deep sequencing the sRNA population in natural conditions, here we provide clear evidence that observed between-species sequence conservations were not always maintained at the within-population level (Figure 4). Errors generated during library construction and sequencing cannot be completely ruled out. However, it is unlikely to be wholly due to the possible PCR and Solexa sequencing errors that both occur at about 10^-5^ per nucleotide [79,80], which is incomparable with the observed mutation rates (e.g. Pi >10%) shown in Figure 4. This surprising observation on high mutation rates at some CSF sites (Figure 4) introduces a new task for RNAi development: to prevent viral escape via population structure shift, i.e., RNAi needs to be designed to target true CSFs rather than those only displayed at higher (between-isolate/between-species) levels. Highly polymorphic sites at the within-population level should be avoided in RNAi design. Otherwise, existing polymorphism in the virus population can mediate rapid escape from designed RNAi [75,81].

Ample evidence shows that VDV-1/DWV/KV clade viruses contribute to the collapse of honeybee colonies in the northern hemisphere (reviewed by [1,2]). It is necessary to develop treatment and control measures against infections by these viruses. Following the trials against the *Israeli Acute Paralysis* (IAPV) in *A. mellifera* [59,60], immunisation by feeding with double-stranded viral RNA has been shown to be an effective approach to reduce DWV load and disease symptoms in honeybees [26]. Desai et al. [26] targeted a 700 nt long fragment (8565-9355 nt, AY292384) at the DWV 3’-end fraction, which contained two CSFs described in Figure 4. Although an effective siRNA profile was not provided to clarify the mechanism(s), RNAi was the primary candidate that mediates the ds-RNA induced immunity [26]. The vsiRNA map shown in Figure 2 supports this speculation, i.e., the vsiRNA against VDV-1/DWV/KV originated from both plus and minus strands and was mainly Dicer products of 21-23 nt long, similarly as characterised for the IAPV RNAi [59,60]. New knowledge on vsiRNA and CSF profiles provided in this study will help to develop more efficient RNAi protocols to combat the virus infections.

DWV and VDV-1 can infect bumblebees and other pollinators [28,32,62,69]. Due to the relatively small sample size used in this work, lack of detection of DWV/VDV-1 in *B* *. pascuorum* cannot be used to conclude that the local infection may be limited to *A. mellifera*. A full picture of the virus population would only be revealed when all major host and vector species are tested for virus. This work in *A. mellifera* demonstrates that virus evolution events like genome recombination are ongoing as well as nucleotide conservation. Virus evolution may lead to emergence of unexpected diseases whereas conservation provides possible targets for developing treatments and control measures.

## Materials and Methods

### Ethics Statement

No permits were required for the described study, which complied with all relevant regulations. The field site is a public place and the study did not involve endangered or protected species.

### Sampling, RNA extraction, and small RNA sequencing

Foraging wild female worker honeybee (*Apis mellifera*) and bumblebee ( *Bombus* *pascuorum* ) individuals were net caught in Sept 2010 whilst foraging on a single large patch of *Succisa* *pratensis* in Bernwood Forest, Oxfordshire, UK. The samples were stored at -80°C until use. The thoraxes of 10 individual *A. mellifera* and *B* *. pascuorum* were pooled, respectively, and extracted for total RNA (>200 nt) and small RNA (<200 nt) fractions using the mirVana miRNA isolation kit (Cat AM1560, Ambion, Life Technologies Ltd, Paisley, UK) following the manufacture’s protocols. Concentrations of the sRNA extracts were measured by using the Nanodrop 1000 UV-Vis spectrophotometer (Nanodrop Products, Wilmington, USA). The two sRNA extracts were quantitatively (1:1) pooled and despatched for small RNA sequencing (at BGI, Hong Kong, China). The deep sequencing dataset has been deposited in the NIH Short Read Archive: accession number SRA069239

### Bioinformatic analyses

Raw sequence reads were subjected to a standard Illumina Solexa quality control pipeline, which also removed the adaptor sequences. The high quality sRNA reads were filtered against the non-coding RNA database (http://biobases.ibch.poznan.pl/ncRNA/). After removing the ncRNAs, the sRNA library was assembled using the SOAPdenovo2 program (http://soap.genomics.org.cn/soapdenovo.html) [63]. Resulting contigs were used to search against the nt/nr database for virus hits using the BLAST program [64]. The sRNA library was then mapped to the NCBI reference sequences of the VDV-1, DWV and KV genomes. After identification of viruses present in the sample, the sRNA library was also used to assemble the virus genomes with the NCBI virus reference sequences (DWV, NC_004830; VDV-1, NC_006494; KV, NC_005876) with the Maq program (Maq 0.6.6, http://maq.sourceforge.net/) [65]. The assembled virus genomes (NCBI accession number: :VDV-1_Ox, KC786222; DWV_Ox, KC786223; KVlike_Ox, KC786224) were aligned with the reference genomes and the other NCBI virus genome sequences to identify conserved regions using ClusterX [66]. Single nucleotide polymorphism (SNP) profiles were identified by using the DnaSP program [67].

### RT-PCR, Cloning and Sanger sequencing

Consensus sequences shared among the assembled genomes were used to design experiments to amplify recombination junction regions. DNA primers were made as F1: 5'-tggagtagatggactagtaatgatgt-3', R1: 5'-aggaacataacctacaattaaccta-3' (synthesized by Eurofins MWG/Operon, Ebersberg, Germany) for amplifying the 5’ recombination junction located at VDV-1 genome positions of nt 1874-3001. A second set of primers was made as F5: 5'-tgacttaacggctgaaatgaatca-3' and R5: 5'-ttcatttcctccactaagcgctgatt-3' for covering the 3’ recombination site at the VDV-1 genome position of nt 5386-6252. The total RNA extracts of honeybee and bumblebee were used as templates for RT-PCR reactions by using the Qiagen one step RT-PCR kit (Cat 210210, Qiagen, Crawley, UK). The reactions were performed on an Omn-E Thermal Cycler (Hybaid Ltd, Cambridge, UK) with a program of 50 °C 30 min, 95°C 2 min; 30 cycles of 95°C 1 min, 55°C 1 min, 72°C 1 min; and 72°C 10 min. The DNA products were examined using 1% Agarose gel electrophoresis, then cloned into the pGEM-T vector plasmid (Cat A1360, Promega, Southampton, UK) and sequenced from both directions using the Big Dye Terminator kit (Applied Biosystems by Life Technologies Corporation, Carlsbad, USA) with the T7 and SP6 primers. Resulting sequences (NCBI Accession No: KC691296-KC691301) were used to construct phylogenetic trees using the Mega5 program [68].

## Supporting Information

Figure S1 **Reads length distribution.**
(PDF)

Figure S2 **Percentage of reads matched to non-coding RNAs.**
(PDF)

Figure S3 **Count of reads aligned to DWV, VDV-1 and KV.**
(PDF)

Figure S4 **RT-PCR amplification of viral fragments.**
(PDF)

Figure S5 **Neighbour Joint consensus tree of Iflavirus genomes.**
(PDF)

Figure S6 **Site mutations in conserved short fragments (CSF) among DWV, VDV-1, and KV.**
(PDF)

Table S1 **Non coding RNA classification.**
(PDF)

Table S2 **Number and percentage of reads mapped to the three reference sequences.**
(PDF)

Table S3 **Mutation rate (%) of nucleotide positions in conserved short fragments.**
(XLSX)

Text S1 **Contigs matched to genome sequences of DWV, VDV1 and KV.**
(TXT)

Text S2 **CLUSTAL X (1.8) multiple sequence alignment of the 5'-end of the recombination junction (F1_6Seq).**
(TXT)

Text S3 **CLUSTAL X (1.8) multiple sequence alignment of the 3'-end of the recombination junction (F5_7Seq).**
(TXT)
