# Supplementary material for: Sequence Recombination and Conservation of Varroa destructor Virus-1 and Deformed Wing Virus in Field Collected Honey Bees (Apis mellifera)
Source: PLoS One. 2013 Sep 18;8(9):e74508. doi: 10.1371/journal.pone.0074508 (PMC3776811; doi:10.1371/journal.pone.0074508)
Supplement: Figure S1 — Reads length distribution. (PDF) [file pone.0074508.s001.pdf]

Figure S1: Reads length distribution

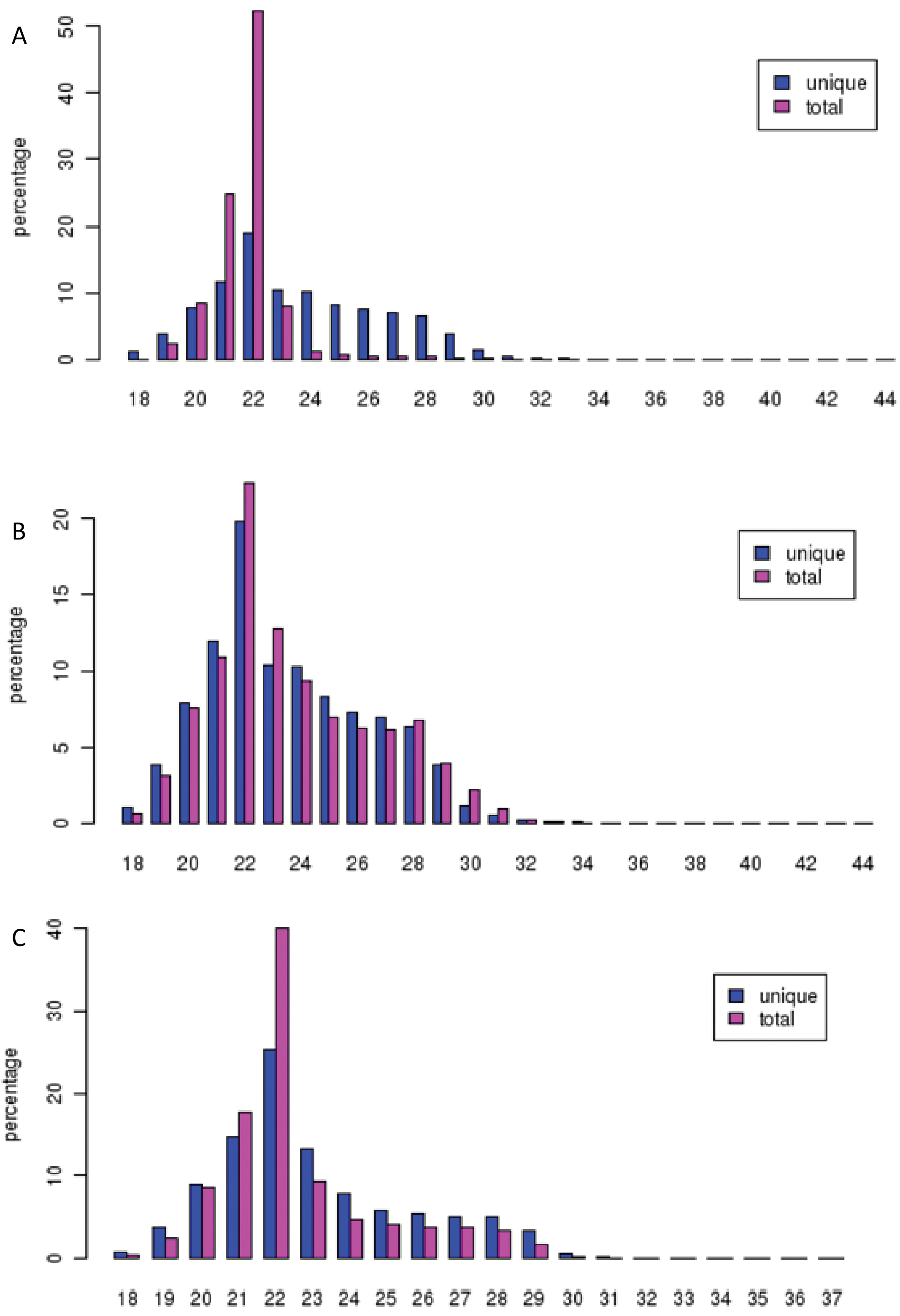

Figure S1: Reads length distribution

A. Original reads
B. After ncRNA removal
C. Virus derived siRNA
