# Supplementary figures and images for: Sequence Recombination and Conservation of Varroa destructor Virus-1 and Deformed Wing Virus in Field Collected Honey Bees (Apis mellifera)
Source: PLoS One. 2013 Sep 18;8(9):e74508. doi: 10.1371/journal.pone.0074508 (PMC3776811; doi:10.1371/journal.pone.0074508)

Figure S2: Percentage of reads matched to non-coding RNAs

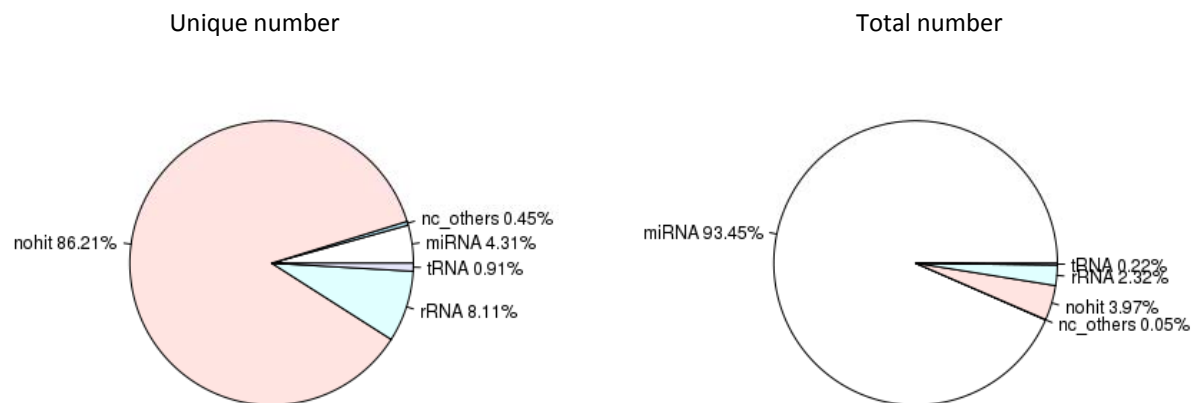

Supplement: Figure S2 — Percentage of reads matched to non-coding RNAs. (PDF) [file pone.0074508.s002.pdf]
