# Supplementary material for: Sequence Recombination and Conservation of Varroa destructor Virus-1 and Deformed Wing Virus in Field Collected Honey Bees (Apis mellifera)
Source: PLoS One. 2013 Sep 18;8(9):e74508. doi: 10.1371/journal.pone.0074508 (PMC3776811; doi:10.1371/journal.pone.0074508)
Supplement: Figure S3 — Count of reads aligned to DWV, VDV-1 and KV. (PDF) [file pone.0074508.s003.pdf]

Figure S3: Count of reads aligned to DWV, VDV-1 and KV

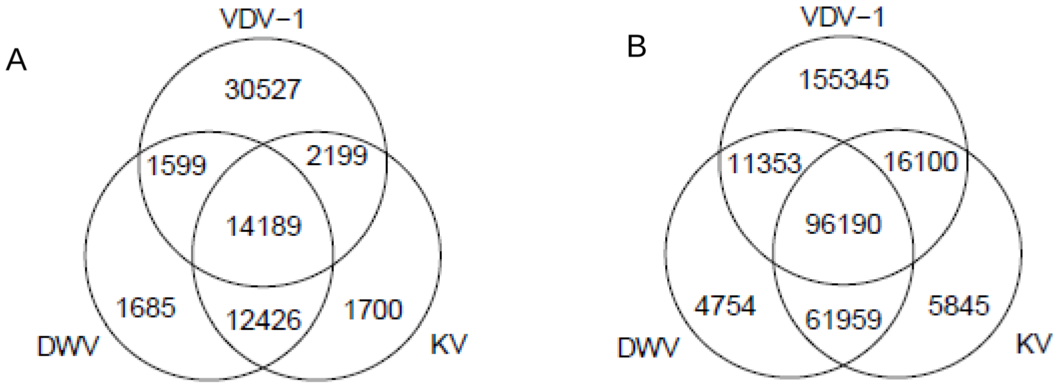

Panel A & B: Counts of reads matched with max mismatch number =2.
(A: unique number, B: total number)

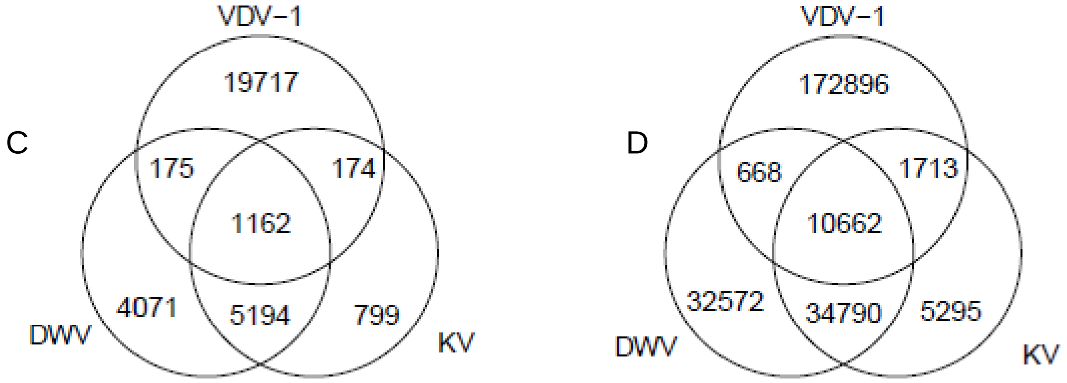

Panel C & D: Counts of reads perfectly matched.
(C: unique number, D: total number)
