# Supplementary material for: Sequence Recombination and Conservation of Varroa destructor Virus-1 and Deformed Wing Virus in Field Collected Honey Bees (Apis mellifera)
Source: PLoS One. 2013 Sep 18;8(9):e74508. doi: 10.1371/journal.pone.0074508 (PMC3776811; doi:10.1371/journal.pone.0074508)
Supplement: Figure S4 — RT-PCR amplification of viral fragments. (PDF) [file pone.0074508.s004.pdf]

Figure S4: RT-PCR amplification of viral fragments

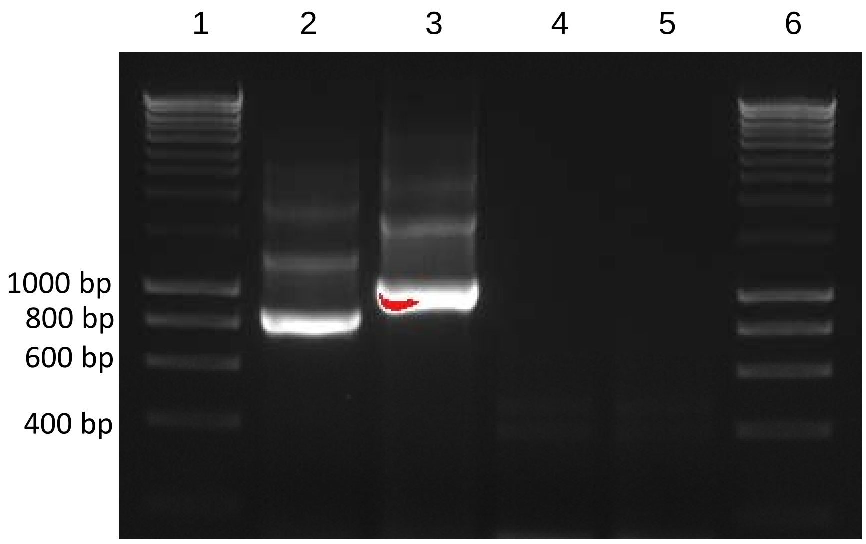

Lanes 1&6: DNA molecular markers (Bioline Hyperladder 1)
Lane 2: Honeybee sample amplified by primers F5&R5. An approximately 850 bp specific product was produced.
Lane 3: Honeybee sample amplified by primers F1&R1. An approximately 1100 bp specific product was produced.
Lane 4: Bumblebee sample amplified by primers F5&R5. No specific product was produced.
Lane 3: Bumblebee sample amplified by primers F1&R1. No specific product was produced.
