# Supplementary material for: Sequence Recombination and Conservation of Varroa destructor Virus-1 and Deformed Wing Virus in Field Collected Honey Bees (Apis mellifera)
Source: PLoS One. 2013 Sep 18;8(9):e74508. doi: 10.1371/journal.pone.0074508 (PMC3776811; doi:10.1371/journal.pone.0074508)

Figure S5: Neighbour Joint consensus tree of Iflavirus genomes.

The tree was constructed using 1000 bootstrap. Triangle labels the VDV-1/DWV/KV clade

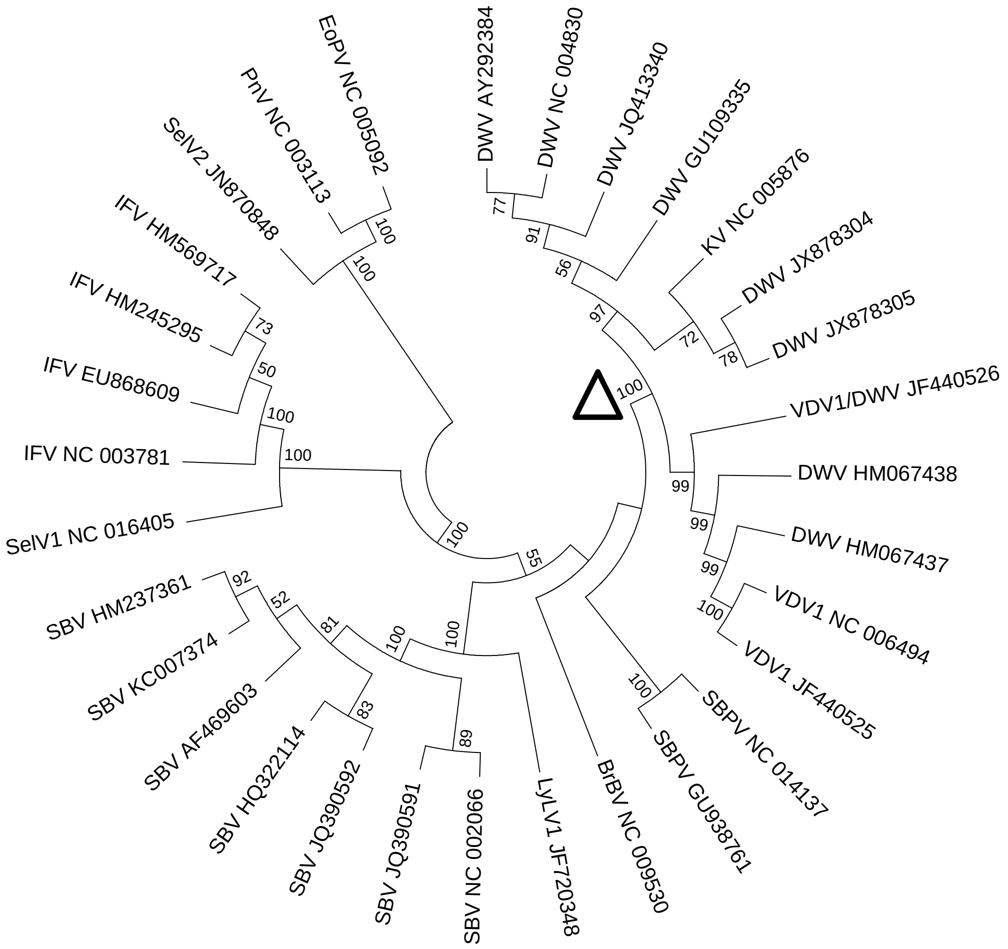

Supplement: Figure S5 — Neighbour Joint consensus tree of Iflavirus genomes. (PDF) [file pone.0074508.s005.pdf]
