# Supplementary material for: Sequence Recombination and Conservation of Varroa destructor Virus-1 and Deformed Wing Virus in Field Collected Honey Bees (Apis mellifera)
Source: PLoS One. 2013 Sep 18;8(9):e74508. doi: 10.1371/journal.pone.0074508 (PMC3776811; doi:10.1371/journal.pone.0074508)

Figure S6: Site mutations in conserved short fragments (CSF) among DWV, VDV-1, and KV

Detected VDV-1/Ox mutation rates of single nucleotide positions (Y-axis) were plotted against sequencing coverage (x1 depth, Panel A), and the positions (5'-3', Panel B) in the CSFs displayed in Figure 4

A

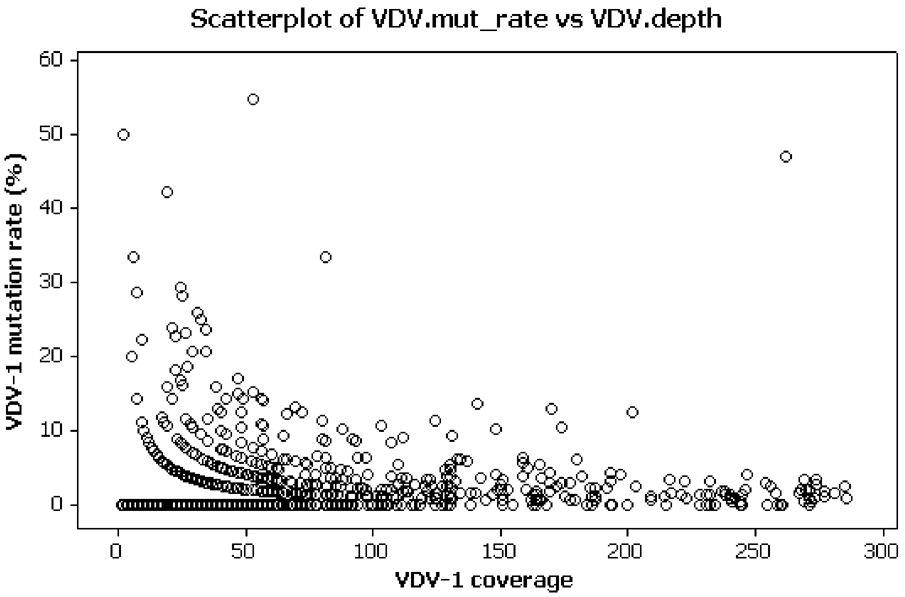

B

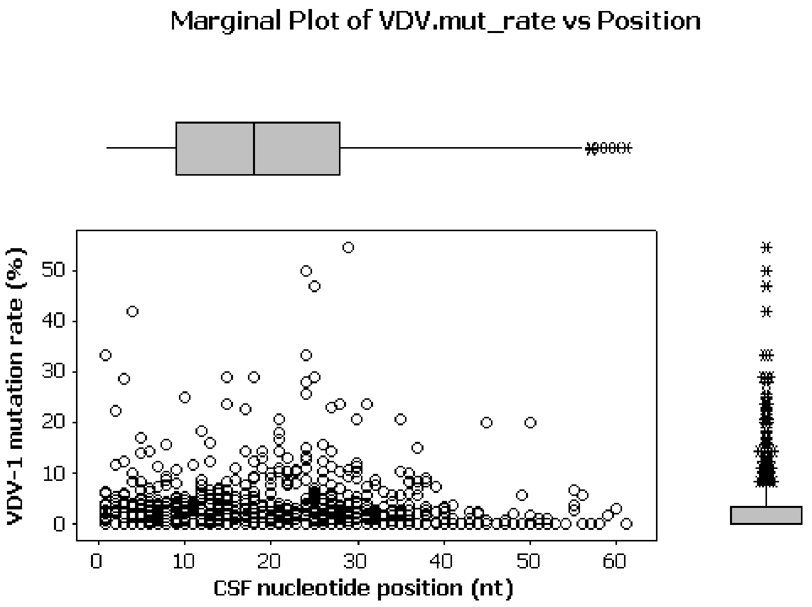

Supplement: Figure S6 — Site mutations in conserved short fragments (CSF) among DWV, VDV-1, and KV. (PDF) [file pone.0074508.s006.pdf]
