# Supplementary material for: Sequence Recombination and Conservation of Varroa destructor Virus-1 and Deformed Wing Virus in Field Collected Honey Bees (Apis mellifera)
Source: PLoS One. 2013 Sep 18;8(9):e74508. doi: 10.1371/journal.pone.0074508 (PMC3776811; doi:10.1371/journal.pone.0074508)
Supplement: Table S1 — Non coding RNA classification. (PDF) [file pone.0074508.s007.pdf]

Table S1: Non coding RNA classification

Item	Total	Total (%)	Unique	Unique (%)
All	94,969,341	100	1,083,543	100
miRNA	88,744,161	93.445	46,741	4.314
tRNA	206,646	0.218	9,890	0.913
rRNA	2,200,499	2.317	87,929	8.115
ncRNA_others	48,991	0.052	4,879	0.45
