# Supplementary material for: Sequence Recombination and Conservation of Varroa destructor Virus-1 and Deformed Wing Virus in Field Collected Honey Bees (Apis mellifera)
Source: PLoS One. 2013 Sep 18;8(9):e74508. doi: 10.1371/journal.pone.0074508 (PMC3776811; doi:10.1371/journal.pone.0074508)
Supplement: Table S2 — Number and percentage of reads mapped to the three reference sequences. (PDF) [file pone.0074508.s008.pdf]

Table S2: Number and percentage of reads mapped to the three reference sequences

	Mismatch<=2		Mismatch=0	
Reference	Unique _num (%)	Total _num (%)	Unique _num (%)	Total _num (%)
NC_004830_DWV	29899(2.76)	174256(0.18)	10602(0.98)	78692(0.08)
NC_005876_KV	30514(2.82)	180094(0.19)	7329(0.68)	52460(0.06)
NC_006494_VDV-1	48514(4.48)	278988(0.29)	21228(1.96)	185939(0.20)
